# Machine learning issues and opportunities in ultrafast particle classification for label-free microflow cytometry

**DOI:** 10.1038/s41598-020-77765-w

**Published:** 2020-11-26

**Authors:** Alessio Lugnan, Emmanuel Gooskens, Jeremy Vatin, Joni Dambre, Peter Bienstman

**Affiliations:** 1 Photonics Research Group, UGent - imec, Technologiepark 126, 9052 Ghent, Belgium; 2 Center for Nano- and Biophotonics (NB-Photonics), Ghent University, Technologiepark 126, 9052 Ghent, Belgium; 3 IDLab, UGent - imec, Technologiepark 126, 9052 Ghent, Belgium; 4 Chair in Photonics, CentraleSupélec and Université Lorraine, LMOPS EA 4423, 2 rue Edouard Belin, 57070 Metz, France

**Keywords:** Imaging and sensing, Biomedical engineering, Computer science, Other photonics

## Abstract

Machine learning offers promising solutions for high-throughput single-particle analysis in label-free imaging microflow cytomtery. However, the throughput of online operations such as cell sorting is often limited by the large computational cost of the image analysis while offline operations may require the storage of an exceedingly large amount of data. Moreover, the training of machine learning systems can be easily biased by slight drifts of the measurement conditions, giving rise to a significant but difficult to detect degradation of the learned operations. We propose a simple and versatile machine learning approach to perform microparticle classification at an extremely low computational cost, showing good generalization over large variations in particle position. We present proof-of-principle classification of interference patterns projected by flowing transparent PMMA microbeads with diameters of ${15.2}\,\upmu \text {m}$ and ${18.6}\,\upmu \text {m}$. To this end, a simple, cheap and compact label-free microflow cytometer is employed. We also discuss in detail the detection and prevention of machine learning bias in training and testing due to slight drifts of the measurement conditions. Moreover, we investigate the implications of modifying the projected particle pattern by means of a diffraction grating, in the context of optical extreme learning machine implementations.

## Introduction

Flow cytometers are instruments able to analyze and characterize large numbers of suspended biological cells and microparticles one by one, while these are flowing at high speed through a measuring device^1^. In traditional flow cytometers, the moving particles are illuminated, usually by a laser, and the corresponding forward and/or side-scattering intensities are measured, together with the fluorescent emission of selectively attached probes. These devices are widely used to investigate the structure and the chemical composition of large populations of cells in many applications concerning life science and clinical diagnosis. Moreover, they also find diverse applications in industrial and environmental engineering fields, e.g. in measuring bacteria viability^2^ or water quality^3^.

Although flow cytometers were constantly innovated upon in the last few decades, their usage is still limited by high cost, complexity and size^4^. Let us now follow a path through some of the recent approaches proposed by the scientific and engineering community to overcome these limitations, in order to contextualize the presented work.

To begin with, the integration of microfluidic systems on a chip allows for a great reduction in cytometers’ cost and size, which is particularly appealing for point-of-care applications^4^. Furthermore, the integration with other lab-on-chip devices provides the opportunity for increased automation and for scalable parallelization of particle analysis, potentially multiplying the overall device throughput^5–7^. While the use of fluorescent labels in microflow cytometry provides a powerful instrument to discriminate between different cell populations at high throughput (even exceeding 100, 000 cells/s^6^), the application of fluorescent stains (also called labels) often hinders live cell analysis, e.g., because of cytotoxicity and requires dedicated effort and cost^8^. Two increasingly common approaches to enable accurate and relatively fast label-free analysis while improving detection sensitivity are given by *electrical impedance detection* and *imaging flow cytometry*^4^. This work mainly focuses on the latter, whose main advantage is the acquisition of detailed spatial information that can be used both for morphology-based detection and for human visualization as in traditional microscopy. On the other hand, the operational speed of camera-based cytometers is limited by the acquisition frame rate, providing single-channel throughputs up to around 1000 cells/s when single cells are captured^9^. This limitation can be overcome, at the cost of increasing system and instrumentation complexity, by encoding optical spatial information into a temporal sequence that is measured by a single photodetector. An application of this technique, named Serial Time-Encoded Amplified Microscopy (STEAM), combines the wide spectral bandwidth of a femtosecond pulse laser with both temporal and spatial dispersive optical elements achieving label-free single cell imaging at a very high throughput, up to $\sim 100,000$ cells/s^10–12^.

The automatic analysis of digital images is a powerful and versatile tool, but it is usually computationally expensive and memory hungry due to the high data dimensionality given by the number of pixels. In high-throughput imaging cytometers, the huge number of stored images and the required processing time are an issue^9^, even more when compact and cheap applications are targeted, e.g. point-of-care. Furthermore, online image analysis often requires a too high computational power such that real-time cell sorting cannot easily be done. Several machine learning approaches have recently been proposed to automatically analyze the big amount of data generated by label-free imaging flow cytometry^8,13–19^, although in most of them the image processing is carried out offline. Exceptions are^15,16,20^, where single-particle classifications respectively took $< 1 \,\text {ms}$, 0.2 ms and 3.6 ms when accelerated by a GPU. These were applied on images of respectively 21 $\times$ 21 and 32 $\times$ 32 pixels in the first 2 works, while in the third the original time-stretch-microscope resolution (which was not explicitly mentioned) was reduced by a factor of 40. However, these execution times are still far from enabling real-time classification for state-of-the-art high throughputs of around 100, 000 cells/s, especially if higher resolutions are required to distinguish specific cell features.

The employment of lensless microscopy constitutes a further step towards significantly cheaper and more compact imaging flow cytometers^17,21^. Since in these devices there are no hardware focusing components, an image reconstruction is performed in software, usually taking from few tenths of a second to several seconds depending on the algorithm or image resolution^16,22^. The idea of bypassing the computationally expensive image reconstruction and performing the machine learning classification directly on the acquired interference pattern was proposed in the past^23^ and recently experimentally applied^16^.

In this work, we present an experimental proof-of-principle study of some key machine learning issues and opportunities regarding fast particle classification with label-free imaging flow cytometry. To do so, we employ a lensless microflow cytometer for real-time label-free particle classification in its minimalist form, both in terms of components and of computational cost. Including a simple visible laser, a pinhole, a microfluidic channel with pumping mechanism and a camera, it only requires a weighted sum of the pixel values to classify a particle from its background-subtracted 2D interference pattern. A simple-to-train machine learning linear classifier (logistic regression) is employed, which does not require any feature extraction based on domain knowledge. In spite of their simplicity, linear classifiers can be as powerful as other state-of-the-art classifiers when applied to high-dimensional representations of input data (a 2D interference pattern in this case). *Extreme Learning Machines*^24,25^ (ELM) and *Reservoir Computing*^26,27^ (RC) are two widespread machine learning approaches based on this principle, employed for time-independent and for time-dependent processing respectively. Indeed, complex classification tasks, such as separation of cell types with similar morphology, can be in principle improved by simply interposing proper optical diffractive layers between the microfluidic channel and the camera^28^, without increasing the classification time. Therefore, we also demonstrate a method to appropriately evaluate the change in classification performance when interposing a diffraction grating, which can be directly generalized to the interposition of other arbitrary diffractive layers, setting the ground for hardware-based improvement of the proposed classification technique.

In this paper, we also place a special emphasis on detecting and preventing a particularly deceptive and often underestimated type of overfitting (called here *measurement bias*), which occurs when the influence of the experimental conditions on the training samples is exploited by a machine learning model to wrongly learn how to carry out a classification task. If the samples used to test the classification performance are biased by the measurement conditions in a similar way, a traditional cross-validation would generally fail in detecting the problem and would instead provide misleadingly high performance evaluations. A machine learning-based cytometer employed for particle classification is likely to be affected by measurement bias when, during the training samples acquisition, the particles belonging to different classes are not mixed but are analysed at different times. Nevertheless, this option is often preferable in practice, because it allows to avoid including a dedicated and accurate ground truth provider system (e.g. based on fluorescent labels detection) in the cytometer. In this work we propose and demonstrate a training and validation approach that allows to detect and prevent such a measurement bias.

For our proof-of-principle study, we consider the classification of PMMA microparticles with different diameters: $(15.2 \pm 0.5) \upmu \text {m}$ (class A) and $(18.6 \pm 0.6)\upmu \text {m}$ (class B), where the error is given by the nominal standard deviation of the particle diameter. In the “Results” section, we first describe the main measurement and machine learning aspects. After that, our method to detect and prevent measurement bias is presented and demonstrated. The classification results obtained for different fields of view of the cytometer and for different image resolutions (including execution time evaluation) is discussed. We also study the effect of interposing different diffractive layers between the camera and the microfluidic channel. Additionally, we compare the obtained results with the ones presented in 3 other relevant works. In the “Discussion” section we summarize the work and discuss the general conclusions. The “Methods” section is dedicated to the technical details.

## Results

### Interference patterns acquisition and machine learning classification

**Figure 1:**
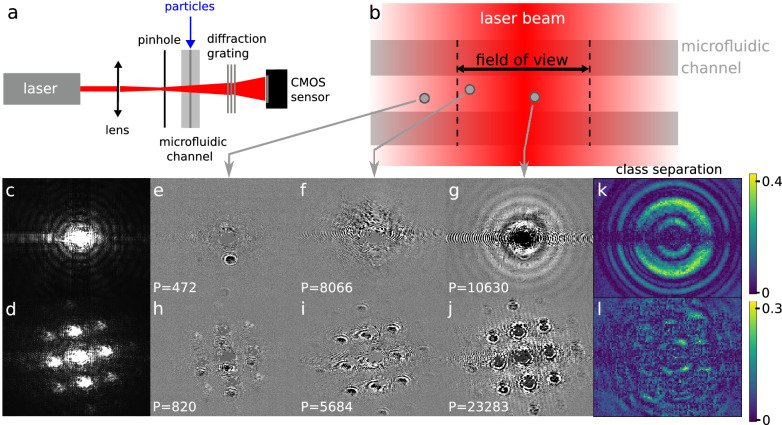
(**a**) Schematic of the employed setup. A PMMA microfluidic channel (cross section ${100}\,\upmu \text {m} \times {100}\,\upmu \text {m}$) is illuminated by a laser radiation (HeNe laser, $\lambda = {632.8}\,\text {nm}$) focused on a pinhole. The resulting beam passes through a double axis holographic diffraction grating (only in one of the employed configurations) and is captured by a CMOS camera. (**b**) Schematic of the illuminated microfluidic channel region. The larger the particle distance from the field of view center, the weaker the acquired particle signal (measured by the perturbation quantity *P*). (**c**–**l**) Respectively for the *NDG* (top row) and *DG* (bottom row) configurations, examples of background pattern (1st column), background-subtracted particle patterns with increasing intensity (2nd to 4th columns) and class separation colormaps (last column). (**e**,**h**) are well below the respective acceptance thresholds, in this case $\theta _P^{NDG}\sim 7200$ and $\theta _P^{DG}\sim 5100$ (for a particle ratio $R=0.04$). (**f**,**i**) are just above and (**g**,**j**) are well above the respective acceptance thresholds. Grey arrows suggest a qualitative link between these examples and the particle position w.r.t. the FoV shown in (**b**).

Employing a CMOS image sensor, we acquired the interference patterns obtained by shining red laser light on transparent PMMA microparticles (with diameters of $(15.2 \pm 0.5) \upmu \text {m}$ and $(18.6 \pm 0.6)\upmu \text {m}$) flowing in a ${100}\,\upmu \text {m} \times {100}\,\upmu \text {m}$ microfluidic channel (Fig. 1a,b). We also performed some measurements interposing a double axis holographic diffraction grating between the microfluidic channel and the camera to modify the imaged pattern (see subsection “Classification performance when mixing with diffractive layers”). The setup configuration with *no diffraction grating* interposed will be referred to as *NDG*, while the configuration comprising the *diffraction grating* will be referred to as *DG*. Figure 1c,d show examples of the acquired background pattern for the two configurations.

**Figure 2:**
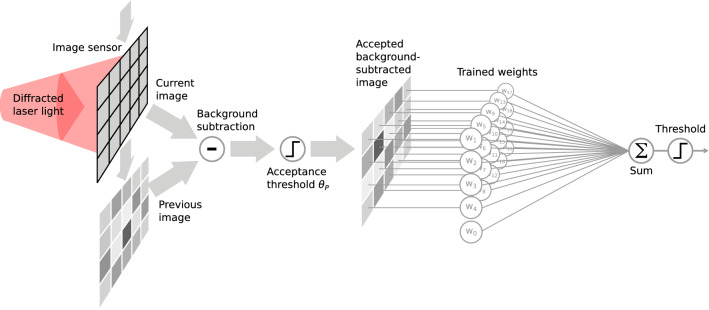
Schematic of the machine learning classification pipeline. Intensity patterns are acquired by the image sensor in free-run mode. The difference between consecutive images is calculated (background subtraction), and if the squared sum of its pixels is lower than a chosen acceptance threshold value $\theta _P$ the image is considered as background and discarted. A linear classifier (trainable weighted sum) is applied to accepted background-subtracted images. If the outcome is positive, the analyzed particle is classified as belonging to class A, to class B otherwise.

The classification process is schematized in Fig. 2. We performed background subtraction on each image by subtracting the previously acquired one. (Because of our flow rates, the probability of having two consecutive frames containing significant particle signal is low.) To ensure that the background subtraction did not introduce any significant artificial particle signal in the sample set, we discarded those images that directly followed an accepted one (image “acceptance” is described in the following lines). Since the CMOS sensor operated in a free-run mode, many of the acquired images contained the background illumination pattern without particles or with only a weak signal from particles far away from the illumination center. Instead of considering these unimportant images as an additional class for the machine learning classifier, we chose the simpler option of discarding them. To do this, we needed to measure the strength of the particle signal: for each background subtracted pattern we calculated the sum of all the squared pixel values, which from now on will be referred to as *overall perturbation* *P*. Examples of background subtracted images with the respective *P* values are shown in Fig. 1e–j respectively for the *NDG* and for the *DG* configurations. Only those images whose *P* value is larger than a chosen *acceptance threshold* $\theta _P$ were accepted as samples used to train and test the machine learning classification. The criteria and the motivation for the choice of $\theta _P$ will be explained in detail later on in this article. Finally, it should be stressed that the particle class could not be straightforwardly determined by human examination.

Similarly as in^28^, in this work we trained and tested a simple linear classifier based on *logistic regression*, directly applied on the pixel values of background-subtracted images. Its task was to classify the acquired interference patterns according to the microbead diameter. We employed *L2 regularization* to reduce overfitting and we optimized its strength by means of k-fold cross-validation, with number of folds $N_s = 11$ (see next subsection for details). When images with high resolutions ($> 10,000$ pixels) were employed as classification samples, a feature selection procedure was applied to reduce both the risk of overfitting and the training time. In particular, we discarded those pixels that showed low *class separation*, i.e. where the value distributions corresponding to the considered classes showed a small difference (see “Feature selection” in the “Methods” section for more details). The calculated class separation can also provide interesting insight on which areas of the acquired interference pattern are most relevant to the classification purpose (Fig. 1k,l).

### Effects and prevention of measurement bias

Supervised machine learning algorithms can learn how to carry out a certain task on a given sample population, e.g. classification of cells in digital pictures, by analyzing a set of training samples for which the solution to the task (i.e., the training label) is given. Therefore, the performance of such algorithms when applied on unseen samples (generalization) is obviously limited by how comprehensively the training samples represent the target sample population. When the noise in the samples and the labels are uncorrelated, generalization can be usually improved by increasing the number of training samples or through regularization techniques, i.e. reducing the overfitting. This is a very well-known practice and in this case the presence of overfitting can be easily detected by testing the algorithm on samples that were not used in the training stage. Less known and more deceptive is the case where the noise and the training labels are correlated, e.g. in classification problems where samples from different classes are acquired or measured under significantly different experimental conditions. In this case, which we will be referring to as *measurement bias*, the machine learning training is most likely biased by the measurement conditions, which are mistakenly considered as a distinguishing trait of the classes. This leads to a worsening of the classification performance under new measurement conditions, i.e. to a decrease in generalization. The elusiveness lies in the fact that measurement bias leads to misleadingly high estimated accuracies and cannot be detected if the training and test samples are measured under the same biasing conditions.

To apply this more concretely to our case of an imaging microflow cytometer, e.g. to train a label-free white blood cell classifier, for practical reasons, monocytes and granulocytes might be kept separated and their images (used as training and test samples for the machine learning algorithm) might be acquired in different measurement sessions, often leading to measurement bias because of drift in between sessions. Indeed, many factors may produce significant drifts in measurement parameters, such as fluctuations of the light source properties, displacement or distortion of the optical beam (e.g. due to thermal expansion of some elements), refractive index changes of the optical components (e.g. due to slow water absorption of the microfluidic channel walls) and so on.

It should be stressed that in this case background subtraction might mitigate but cannot completely remove the measurement bias, as it is demonstrated in the next paragraph. Indeed, the background signal is given by the unperturbed laser beam impinging on the camera screen while the particle signal is mainly given by a spatial optical path perturbation of the same laser beam. These two signals are combined in a strongly nonlinear way by the image sensor measurement and therefore they cannot be decoupled by a simple linear operation such as background subtraction.

Another approach to remove measurement bias is to mix the two kinds of cells and determine their class (i.e. their label) during the image acquisition using an auxiliary system, e.g. a fluorescent label detector. However, including such a system is more complex, also considering that to train an accurate classifier even more accurate ground truth data is required. This is therefore not what we considered in this paper.

**Figure 3:**
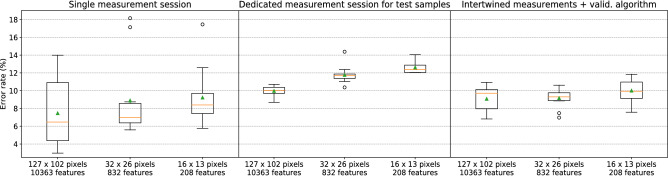
Box plots of the classification error evaluated by means of cross-validation on images down-sampled to different resolutions (x axis). Each box represents the distribution of the $N_s$ error values, corresponding to different folds, obtained through k-fold cross-validation. Boxes, whiskers, orange lines and green triangles respectively represent the interquartile range, the range, the median and the mean of the error values. The outliers (outer points distant more than $1.5\times$(interquartile range) from the interquartile range) are represented by circles. The employed samples were selected among the acquired images considering a particle ratio value of $R = 0.04$ (see subsection “Microbeads classification for different fields of view”). *Left*: the samples employed for training, validation and test were obtained from a single measurement session per class, providing misleadingly low average errors and high variance due to measurement bias. *Middle*: test errors are evaluated on samples from dedicated measurement sessions, showing the correct generalization capability of the trained classifier. *Right*: the proposed intertwined class measurements and validation algorithm were employed to remove the measurement bias influence from classification training, validation and test. The comparison with the middle box plot shows an improved generalization capability of the trained classifier.

In order to provide an experimental demonstration of the negative effects of measurement bias, we performed ad hoc sample measurements according to the following chronology:

1 $$\begin{aligned} A_{train}(\text {20 mins}),\, B_{train}(\text {20 mins}),\, A_{test}(\text {2 mins, 15 s}),\, B_{test}(\text {2 mins, 26 s}) \end{aligned}$$

where *A* and *B* refer to interference patterns acquisition of PMMA beads with diameter of $15.2 \,\mu m$ and $18.6 \,\mu m$ respectively. Even employing a proper cross-validation technique, using samples from $A_{train}$ and $B_{train}$ for training, validation and test, the employed particle classification provides on average significantly lower test errors than when $A_{test}$ and $B_{test}$ are employed for testing (Fig. 3, compare *left* with *middle*). This means that the classifier training was influenced by the measurement conditions leading to an overestimated generalization capability when samples from the same measurement session were employed for testing. Such an effect is also responsible for a large variance in performance evaluation ascribed to the fluctuations of the measurement conditions during the measurement sessions.

In this work we developed a simple method to solve this problem, i.e. to effectively decouple the training sample labels from slow fluctuations of the measurement parameters, avoiding measurement bias. In particular, we acquired the samples according to the following measurement sessions chronology (duration of 2 mins each):

2 $$\begin{aligned} A_1,\, B_1,\, A_2,\, B_2,\, \ldots \, , A_{N_s}, B_{N_s} \end{aligned}$$

i.e. using *intertwined class measurements* to provide training, validation and test samples to the classification algorithm. In all cases, the measurement sessions were performed at different times in the same day. Considering a number of sessions per class $N_s = 11$, we then employed the following *validation algorithm*:

3 $$\begin{aligned} {} & {} \text {for i} = 1,2,\ldots , N_s: \\&\qquad A_{train}^i \leftarrow \bigcup _{n\ne i} A_n \quad , \quad B_{train}^i \leftarrow \bigcup _{n\ne i} B_n \\&\qquad \text {for j} = 1,2,\ldots , N_s - 1: \\&\qquad \qquad A_{train}^{ij} \leftarrow \bigcup _{n\ne i,j} A_n \quad , \quad B_{train}^{ij} \leftarrow \bigcup _{n\ne i,j} B_n \\&\qquad \qquad \text {for k} = 1,2,\ldots , N_h: \\&\qquad \qquad \qquad \theta _{ijk} \leftarrow \text {train classifier}(A_{train}^{ij},\, B_{train}^{ij}\, | \, h_k) \quad , \quad p_{ijk} \leftarrow \text {test classifier}(A_j,\, B_j\, | \, \theta _{ijk},\, h_k) \\&\qquad \tilde{h}_i \leftarrow \text {select best hyperparameter}(p_{ijk}) \\&\qquad \theta _{i} \leftarrow \text {train classifier}(A_{train}^{i},\, B_{train}^{i}\, | \, \tilde{h}_i) \quad , \quad p_{i} \leftarrow \text {test classifier}(A_i,\, B_i\, | \, \theta _{i},\, \tilde{h}_i) \\&p_{final} \leftarrow \text {average}(p_i) \\ \end{aligned}$$

where $h_k$ is a hyperparameter (L2 regularization strength in our case) to optimize by choosing among given options corresponding to $k = 1,2,\ldots ,N_h$ and $\tilde{h}$ is the chosen hyperparameter value; $\theta$ is the set of readout parameters (weights and intercept) determined by the training, *p* refers to a performance evaluation (the estimated accuracy in this case) of the machine learning classifier and $p_{final}$ is the final evaluation of the whole algorithm, including the hyperparameter selection. The generalization of the algorithm to multiclass and multiple hyperparameters cases is straightforward. The main concept here is that the training, validation and test datasets not only are always disjoint as it happens in traditional cross-validation, but they were also acquired in different and chronologically separated measurement sessions. Even though it should be considered good practice, this methodology is not always implemented and in this work we show some possible misleading consequences.

Applying the proposed intertwined measurements and validation algorithm, we obtained better classification performance (Fig. 3, compare *right* with *middle*). Moreover, we obtained an evaluation of the accuracy average and variance generalized to different measurement sessions. As explained in the next subsection, we checked if the measurement bias was still affecting our results by means of a suitable test (UM test). The number of sessions per class $N_s$ should be chosen high enough to ensure that the measurement bias is removed and to achieve a satisfactory generalization capability of the trained classifier. Generally, $N_s$ is limited by the difficulty and the time required to perform a high number of measurement sessions to provide training samples. Therefore, an optimal $N_s$ is highly application-dependent.

### Classification performance vs. field of view

According to how displaced the flowing particle is w.r.t. the laser beam center, the acquired interference patterns may vary in intensity, position and shape (e.g. Fig. 1c,e,f), making the particle analysis more or less difficult. The range of such displacement for which it is still possible to perform the particle classification is called *field of view* (FoV) of the cytometer. In this case, the time interval between two consecutive image acquisitions is much longer than the travel time of a particle through the FoV, implying that a fraction of the flowing particles are not measured. Thus, the larger the FoV the higher the number of particles that are analysed w.r.t. the total number of flowing particles and therefore the higher the maximum *sensitivity* of the cytometer. Usually, the sensitivity of particle detection can be enhanced by employing an effective microfluidic focusing system^4^, even though there is a trade-off between fabrication complexity, sensitivity and throughput. In any case, the particle displacement along a microfluidic channel always constitutes an important source of variability.

In this work, we estimated the classification performance considering different unidimensional FoV values along the microfluidic channel direction. The transverse channel dimensions were neglected since the illumination was considered to be relatively uniform on the channel cross sections. As it is intuitively schematized in Fig. 1b, the larger the distance of a particle form the illumination center, the smaller the *P* value of the obtained image. This implies that the FoV is determined by the choice of the acceptance threshold $\theta _P$. Still, two particles belonging to different classes and in the same position will lead to two images with different *P* value. Therefore, in order to have the same FoV for different classes of particles, the applied $\theta _P$ should ideally be class-dependent. However, this is only feasible in the training stage, where the classes (labels) are known, while in the test stage a common acceptance threshold has to be used for all the acquired images. Since this mismatch between training and test sample populations may be detrimental for classification performances, in this work we chose to use a common $\theta _P$ for the two classes in both training and test. In practice, the applied acceptance threshold $\theta _P$ was chosen so that a desired value for the *particle ratio* *R*, defined as the ratio of the number of accepted particle images to the total number of acquired images, is obtained. The reason is that the particle ratio can be used as a more objective bridge quantity in the classification comparison with the cases where diffractive optical layers are interposed between the microfluidic channel and the camera (this is explained in the subsection “Classification performance when mixing with diffractive layers”). For each value of *R*, the FoV for each class can be estimated (see “Calculation of acceptance threshold and field of view given a chosen particle ratio” in the “Methods” section).

**Figure 4:**
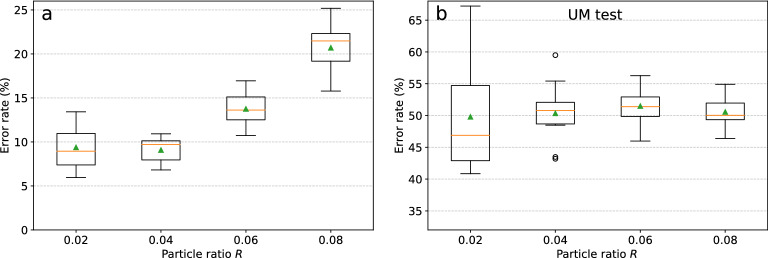
(**a**) Box plot of the classification error evaluated by the proposed validation algorithm on sample sets obtained through different choices of *R* (on the x axis), i.e. applying different acceptance thresholds. $R=0.04$ provides the best classification performance (low error average and variance) due to a trade-off between the field of view and the number of samples *N*. (**b**) Corresponding classification error obtained through the proposed UM test. The training is performed on uniformly mislabelled data and therefore the obtained test error is expected to be $\sim 50\%$ (random choice) for our two classes, if the learning is not affected by measurement bias.

We evaluated the performance of the presented classification algorithm considering sample sets obtained through different choices of $R = 0.02,\,0.04,\,0.06,\,0.08$ (Fig. 4a). The employed image resolution is 127 $\times$ 102 pixels, corresponding to a down-sampling with a factor 5 w.r.t. the camera resolution. A feature selection algorithm (see “Feature selection” in “Methods” section) was applied to remove the most noisy pixels and therefore to decrease the risk of overfitting, leaving a total of 10363 features, i.e. $\sim 80\%$ of the pixels.

The sample set corresponding to $R=0.04$ provides the best classification performance (low error average and variance) due to a trade-off between the quality and the number of samples. Indeed, a lower *R*, or equivalently a higher acceptance threshold $\theta _P$, means we only keep the samples with the highest quality in the center of the laser beam, reducing the FoV. This results in a lower sample variability (which should make the classification easier), but also in a lower number of available samples (which makes it more difficult to train the classifier). It should be stressed that the optimal *R* value is application-specific. In particular, *R* should be chosen so that the classification accuracy is maximized, while trying to achieve the target cytometer throughput. Moreover, the number of available training samples and the classifier complexity (e.g. given by the image resolution) play two major roles in the choice of *R*, because of the need to avoid overfitting.

We furthermore double-checked whether the classifier would still be biased by the measurement conditions, in spite of our intertwined class measurements. This was done by training it on the same dataset but with half of the measurement sessions mislabeled, i.e. in list (Eq. 2): $A_2 \rightarrow B_2$, $B_2 \rightarrow A_2$, $A_4\rightarrow B_4$, $B_4\rightarrow A_4$, and so on. In this way, the characteristic features given by the different sizes of the beads (corresponding to the true classes) were equally present in both the nominal classes (those presented to the training algorithm). Thus, if the classifier only learns the particle-related features and therefore it is not biased, it would provide the same accuracy of a random guess ($\sim 50\%$ in the two-classes case). This *uniform mislabelling* (UM) test shows indeed errors around $\sim 50\%$ in Fig. 4b, which indicates that no significant bias is detected.

### Classification performance and time vs. image resolution

In imaging flow cytometry, the resolution of the acquired images is a key parameter, not only because of the obvious relation with the price and the frame rate of the employed image sensor, but also because it greatly influences the execution time of the particle analysis/classification and therefore the throughput limit of online operations, such as cell sorting.

We evaluated the performance of our particle classification technique for different resolutions of the employed images and we estimated the corresponding execution (inference) times. Different sample sets were obtained downsampling the acquired images by approximated factors 2, 5, 10, 20, 40, 100 and 400. Thus, the original resolution of $632\times 508$ pixels was decreased respectively to $316\times 254$, $127\times 102$, $64\times 51$, $32\times 26$, $16\times 13$, $7\times 6$ and $2\times 2$. Note that for the highest two resolutions respectively 87.1% and 20% of the pixels were discarded by means of feature selection (see “Feature selection” in the “Methods” section), in order to limit overfitting and the computational cost of training the classifier. For the remaining resolutions no feature selection was performed, i.e. all the pixel values were employed as features for machine learning.

**Figure 5:**
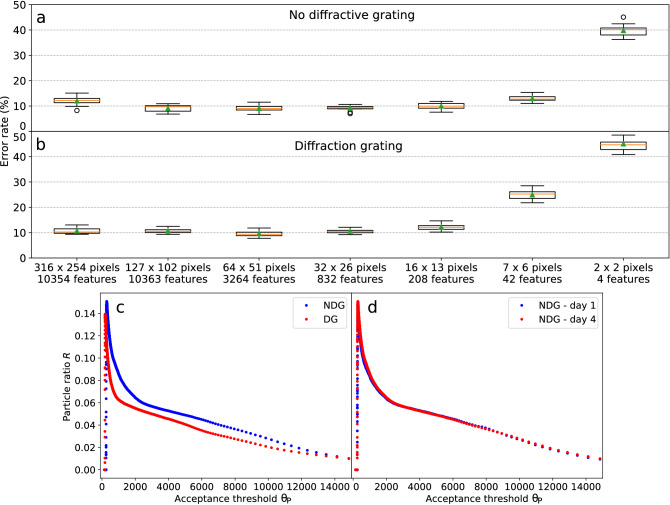
(**a**,**b**) Box plots of the classification error for $R=0.04$ evaluated on particle images of different resolution (x axis), with and without holographic double axis diffraction grating interposed between the camera and the microfluidic channel. Classification errors lower than $10\%$ were obtained for image resolutions down to just $32\times 26$ pixels. Generally, the interference patterns processed by the diffraction grating provide particle classification with similar or slightly higher errors. Note that the number of samples used to evaluate the first two points was further reduced by feature selection. (**c**,**d**) Particle rate *R* as a function of the acceptance threshold $\theta _P$ for different measurement sessions. (**c**) Comparison between the configuration without interposed diffraction grating (*NDG*, blue dots) and with diffraction grating (*DG*, red dots). The diffraction grating changes the relation in a nonlinear way. (**d**) Comparison between measurement sessions (both in *NDG* configuration) performed with a time distance of 3 days. The curve do not change significantly from one measurement session to another, indicating stability in our measurements.

Using the previously determined optimal particle ratio value $R=0.04$, we obtained classification errors below $10\%$ for image resolutions of $127\times 102$, $64\times 51$ and $32\times 26$ pixels (Fig. 5a). The error is just slightly worse using $16\times 13$ pixels, but it abruptly increases for $7\times 6$ and $2\times 2$ pixels, showing that the resolution is too low to provide the classifier with enough particle information. In particular, this shows that the classification task could not be carried out by just considering the total forward scattering intensity, as opposed to bead size discrimination in traditional flow cytometers. This suggests that our classification system presents much less stringent requirements on the alignment of flowing particles with the laser beam. Also selecting $12.9\%$ of the pixels from higher resolution images ($316\times 254$ pixels) leads to a small but significant degradation of the classification performance. Setting $R = 0.02,\,0.06$ or 0.08, similar performance trends with an overall degradation were obtained. It should be stressed that the relation between the classification error and the image resolution depends on the addressed classification task and cannot be generalized.

The average execution time of the classification algorithm inference (i.e. background subtraction + application of acceptance threshold + machine learning inference , see Fig. 2), was evaluated for different image resolutions running a Python script on a normal laptop (Intel Core i5-8250U, 1.60GHz $\times 8$). Ultrafast image classification was achieved with computational times per particle in the order of ${100}\,\upmu \text {s}$ to ${10}\,\upmu \text {s}$ depending on the resolution (Table 1). It should be stressed that these values could be easily further decreased by, e.g., employing multi-core computing, a graphics processing unit (GPU) or a dedicated hardware.

**Table 1:** Execution time per particle of the proposed classification algorithm for different image resolutions, evaluated on a laptop (Intel Core i5-8250U, 1.60GHz $\times$ 8) using a Python script (Numpy library). The reported time values are averaged (median) over 10000 iterations of the following steps: computation of the difference between the target and the background image after conversion to float type matrices; application of the acceptance threshold to the sum of the squared elements of the difference matrix; weighted sum of the difference matrix (i.e. machine learning inference).

Image resolution (pixels)	$316\times 254$	$127\times 102$	$64\times 51$	$32\times 26$	$16\times 13$	$7\times 6$	$2\times 2$
Classification time ($\upmu \text {s}$)	200	38	19	13	10	9.0	8.8

### Classification performance when mixing with diffractive layers

From a machine learning perspective, one might intuitively assume that applying a simple linear classifier on the raw pixel values of an image would generally provide a much weaker classification power w.r.t. common approaches based on feature extraction and deep learning. Actually, linear classifiers and regressors can provide state-of-the-art performance when applied to random high-dimensional nonlinear transformations of the input, as it happens in widespread approaches like *Extreme Learning Machines*^24,25^ (ELM) and *Reservoir Computing*^26,27^ (RC). Indeed, the relation between the optical particle features and the detected interference pattern (input and output) is mathematically nonlinear and the high number of pixels in an image sensor can potentially provide a high-dimensional mapping. Therefore, modulating and controlling the interference pattern projection e.g. through interposed diffraction layers can provide an extremely fast and power-efficient source of computational power, as it was experimentally demonstrated in^29,30^. Moreover, in the past^28^ we numerically demonstrated that random diffractive layers that resemble diffraction grating structures can significantly improve the performance of a linear classifier in non-trivial classification of cell structures. However, by interposing diffractive layers between the particle and the image sensor, the automatic discrimination of particle images from background images is likely to be influenced. In particular, this can modify the cytometer sensitivity and the class balance in the training sample sets. In this subsection we present a method to avoid these issues and to guarantee a valid performance comparison, laying the groundwork for hardware-based improvement of the proposed classification technique.

For the sake of simplicity, we present the comparison between two simple configurations: *no diffraction grating* (*NDG*), and one interposed double-axis holographic *diffraction grating* (*DG*, see Fig. 1), with a line period of $\sim {1.88}\,\upmu \text {m}$. In practice, in order to have a fair comparison, the main issue is how to choose the corresponding acceptance thresholds $\theta _P^{NDG}$ and $\theta _P^{DG}$ to make the two cases comparable. We want to compare both cases for a fixed maximum sensitivity of the cytometer, i.e. when the FoV is the same in both configurations. Generally, the introduction of a diffractive layer changes the intensity of the acquired particle signal in a nonlinear way, so that $\theta _P^{NDG} = \theta _P^{DG}$ or even $\theta _P^{NDG} \propto \theta _P^{DG}$ would lead to different FoVs. However, as we discuss in the calculation of the field of view in the “Methods” section, there is a one-to-one correspondence between the *particle flow rate* $R_f$, the particle ratio *R* and the field of view. If we can guarantee in our experiments that the particle flow rate $R_f$ is constant, the requirement of having a fixed field of view translates to a requirement of having a fixed particle ratio *R*. This allows us to set the acceptance thresholds for both configurations, by looking at the experimentally determined relationship between the particle ratio *R* and the acceptance threshold $\theta _P$ (see Fig. 5c).

We also checked whether the particle flow rate $R_f$ did not change significantly from one measurement session to another, and therefore that the relation between *R* and $\theta _P$ remained constant. This was experimentally confirmed by comparing two measurements in *NDG* configuration performed at significantly distant times (3 days one from another, see Fig. 5d).

Generally, the *DG* configuration provided similar or (in most cases slightly) inferior classification performances w.r.t. the *NDG* configuration (for example compare Fig. 5a,b). We ascribe the higher error rates mainly to the significant intensity attenuation by the diffraction grating, leading to a lower signal-to-noise ratio. This issue, however, can be easily overcome in a more mature cytometer implementation, e.g. by enclosing the system in a box or by screening the sensor with an optical filter to reduce noise due to environmental illumination. The main challenge of the classification task studied here is the variability due to the microbead displacement w.r.t. the illumination center, which can be in principle arbitrarily alleviated by decreasing the cytometer FoV. In that case, we expect that a properly designed diffractive layer may improve the classification performance, especially when the particle types are distinguished by differences in internal structure, such as in sorting of white blood cells^28^.

On the other hand, the fact that the classification performance is not significantly disrupted by the heavy deformation of the particle interference pattern due to the diffraction grating (visual examples are in Fig. 1d,h–j,l), demonstrates the robustness of the proposed cytometer. Indeed, the classifier can be trained without any problem when the acquired images are altered, e.g. by fabrication defects, misalignment or blurring, as long as the particle information regarding the difference between classes is not lost. This is relevant in practice, as motion blur is a common problem in imaging flow cytometry^4^ and it often limits the achievable throughput.

### Comparison with other works

In this subsection we compare the classification performance of our method with the performance presented in other three comparable works, reporting online label-free classification (Table 2). It should be specified that the throughput of our setup is quite low ($\sim 2.7$ classified cells per second for $R=0.04$), since our work mainly focuses on general machine learning aspects of label-free imaging flow cytometry rather than on developing a high-throughput device. We should also stress that it is difficult to estimate and compare the complexity of the respective classification tasks, since not only do the particle characteristics play a crucial role, but also cytometer properties such as the FoV, the presence of an image focusing system or the control of measurement bias.

**Table 2:** Comparison of machine learning-related aspects regarding three other works (reporting online label-free classification via particle imaging) and our work. CNN is the acronym for *Convolutional Neural Network*, while mAP is the abbreviation of *mean Average Precision*.

Classification task	Classifier	Image resolution	Imaging method	Image FoV	Classification performance	Accelerator	execution time / particle	Meas. bias control
Beads with diameters of 7, 10 and ${15}\,\upmu \text {m}$^15^	CNN	$21\times 21$	Microscope	Centered and cropped	$93.3\%$ mAP	GPU	$< 1 \,\hbox {ms}$	Unreported
3 white blood cell (WBC) types^16^	Rand. forest on extracted features	$31\times 31$	Lens-free - raw hologram	Unreported	$96.8\%$ accuracy	GPU	0.2 ms	Unreported
1 WBC type and an epithelial cancer cell^20^	Deep CNN	Unreported	Time-stretch microscope	${25}\,\upmu \text {m}$ along channel	$95.74\%$ accuracy	GPU	3.6 ms	Unreported
Beads with diameters of 15.2 and ${18.6}\,\upmu \text {m}$ (our work)	Linear (log. regression)	$32\times 26$	Lens-free - raw hologram	$\sim {300}\,\upmu \text {m}$ along channel	$> 90\%$ accuracy	None	0.013 ms	Yes

In particular, it should be stressed that a wider FoV not only introduces the challenge of generalizing the classification to a higher variability in particle position, but also implies a smaller contrast of the particle signal w.r.t. the background illumination. In this regard, in^20^ the reported FoV is ${25}\,\upmu \text {m}$, much smaller than what we estimated for this work ($\sim 0.3\,\hbox {mm}$, Table 3). While in^16^ there seems to be no mention of it, in^15^ the FoV is comparable with ours, but the actual machine learning classification is applied on cropped and centered particle images so that the variability in particle position does not complicate the classification. Furthermore, it is interesting to note that our classification algorithm is not specifically built to extract position-invariant features, as opposed to the classifiers used in the other works here described. Finally, a distinguishing trait of this work is that the classifier could learn and operate on images that could not be straightforwardly classified or recognized by human inspection (e.g. see patterns in Fig. 1).

This said, the presented bias-free classification is at least 15 times faster w.r.t. the aforementioned works, even if it is only computed with a common laptop and without GPU acceleration.

## Conclusion

We discussed some important machine learning aspects regarding fast particle classification with label-free imaging flow cytometry. To do so, we employed a simple, cheap and compact cytometer and demonstrated ultrafast classification of particle interference patterns, which can enable online high-throughput analysis (e.g. for cell sorting) at a low computational cost. Proof-of-principle experiments were performed by acquiring and classifying interference patterns projected by transparent PMMA microparticles with diameters of $(15.2 \pm 0.5) \upmu \text {m}$ and $(18.6 \pm 0.6)\upmu \text {m}$, that could not be easily classified by human inspection. In particular, we discussed and demonstrated the following fundamental aspects:

- Detection and treatment of the deceptive bias that can affect machine learning models, rising from the correlation between the ground truth information (necessary for training and testing) and the experimental conditions that may influence the measurements.
- Direct application of a linear classifier on background-subtracted images of particle interference patterns, allowing simple and robust machine learning classification of particles with high position variability at an extremely low computational cost.
- A method to properly evaluate the change in classification performance when a diffractive layer (a double-axis holographic diffraction grating film in this case) is interposed between the camera and the microfluidic channel, making sure that the field of view (i.e. the sensitivity) and the class balance of the training sample sets remain unchanged.

A diffraction layer interposed between the camera and the microfluidic channel can in principle improve particle classification according to the Extreme Learning Machine (ELM) paradigm^28^, even though in this case similar or slightly worse performances were achieved. Nevertheless, we think that an experimental demonstration of the classification improvement due to an interposed diffractive layer should be tried by thoroughly exploring different configurations and considering a more morphology-based classification task, such as in white blood cell sorting.

Quantitatively speaking, the best achieved performance in terms of classification accuracy and execution time is an accuracy above 90% (on $32\times 26$ pixels images) with an estimated execution time of ${13}\,\upmu \text {s}$ (using a common laptop) and a field of view of $\sim {300}\,\upmu \text {m}$ along the microfluidic channel. It should be noticed that the accuracy could be enhanced by simply employing a smaller field of view and by acquiring a sufficient number of samples to properly train the classifier. As mentioned, suitable measurements, validation algorithms and tests were devised and employed to obtain a correct training and evaluation of the classification performance, which would otherwise have been biased by slight drifts of the measurement conditions. The proposed particle classification algorithm is at least one order of magnitude faster w.r.t. the state-of-the-art, represented by other three works regarding fast online classification in label-free flow cytometry^15,16,20^, where instead GPU acceleration was employed.

The low computational cost of the proposed classification method could enable ultrafast ($\sim 100,000$ particles/s) online particle analysis if applied to time-stretch microscopy^11,14^, removing or alleviating the issue of storing large amounts of data and allowing fast online operations in these systems, such as cell sorting. Another possible high-throughput application is to perform the cell analysis in parallel employing multiple particle streams, where the computational cost would be a bottleneck parameter^5,7^.

Finally, the all-round simplicity and the low cost of the presented flow cytometry approach make it suitable for compact point-of-care applications, where both the training and the use of the cytometer should not require high technical expertise.

## Methods

### Measurement details

The employed PMMA microbeads mixtures were obtained by diluting the original mixtures ($5\%$ solid content volume) in a solution of water with a small quantity of surfactant and a water purification tablet, reaching a fraction of solid content volume of $0.024\%$. The mixtures were pumped in a ${100}\,\upmu \text {m}\times {100}\,\upmu \text {m}$ straight PMMA microfluidic channel at a constant rate of $\sim 0.003\,\hbox {ml/s}$, using three different syringes (one at a time) respectively for the two particle classes and the flushing water, to avoid particle contamination. Between each measurement session, the microfluidic channel and tubes were flushed with water to remove possible residual microbeads.

The microfluidic channel was illuminated by focusing HeNe laser radiation (constant emitted power of 3.5 mW) on a pinhole (diameter of ${25}\,\upmu \text {m}$) tightly clamped to the microfluidic slide in order to prevent it from moving during measurements and to reduce vibration noise. When employed, the holographic diffraction grating film was directly attached to the front side of the microfluidic slide. A schematic of the employed setup is shown in Fig. 1a. Images of $632\times 508$ pixels were acquired in free-run mode by a Ximea MQ013MG-0N camera, at a frame rate of $\sim 138\,\hbox {fps}$ and with ${29}\,\upmu \text {s}$ exposure time.

### Machine learning pipeline

The whole image processing presented in this work was executed in Python. In particular, the machine learning pipeline was built on top of the *scikit-learn* library^31^ and the following functions were employed: *model_selection.GroupKFold* to implement the two nested cross-validation loops; *preprocessing.StandardScaler* to normalize the features before each training or inference step; *linear_model.LogisticRegression* with “l2” penalty, “liblinear” solver and “balanced” class weight, as linear classifier. The only optimized hyperparameter was the inverse of the L2 regularization strength *C*, chosen among 13 values equidistant in log. scale from $10^{-5}$ to 10. The downsampling to desired image resolutions was performed employing the “block_reduce” function from the *Scikit-image* Python library. The classification error rate reported in the box plots represents the fraction of misclassified test samples w.r.t. the total number of test samples, thus it is the complementary percentage of the classification *accuracy*.

### Feature selection

The feature selection, applied only in the two highest image resolution cases, consists in selecting only a fraction of the pixels, in particular those that show the highest *class separation*. Given a pixel, the class separation tells how stochastically larger or smaller are the values corresponding to one class w.r.t. to the ones belonging to other classes. To obtain a measure for this quantity that is robust against outliers and non-normality, we exploited a simple non-parametric statistic: the *Mann–Whitney U*^32^. In particular, the following normalized (from 0 to 1) expression was considered:

4 $$\begin{aligned} \frac{| U - (n_An_B+1)/2 |}{(n_An_B+1)/2} \end{aligned}$$

where *U* is the aforementioned statistic, calculated through the Python function *scipy.stats.mannwhitneyu* (with the “alternative” parameter set to “two-sided”); $n_A$ and $n_B$ are the number of samples belonging to class A and B respectively. Rather than selecting few important features, the proposed feature selection method is more suited to discard unimportant noisy features from a large set, such as pixels that do not contain particle information, in a computationally cheap and statistically robust way. Moreover, visualising the class separation colormap may provide interesting insight on the interference pattern areas that are most class-dependent (Fig. 1g,n).

### Calculation of acceptance threshold and field of view given a chosen particle ratio

The relation between particle ratio *R* and acceptance threshold $\theta _P$ was graphically obtained by plotting the count of accepted images divided by the total number of images for many values of $\theta _P$ (Fig. 5c). It was then straightforward to select an acceptance threshold corresponding to a chosen particle ratio.

The field of view (FoV) can be derived from the acceptance threshold, knowing the aforementioned *particle flow rate* $R_f$, the *exposure time* $\tau$ and the *fluid velocity* *v*. In particular, let us start by finding the probability that an image contains enough particle information, i.e. that a particle is at least partially present in a given FoV during an exposure time interval $\tau$. Let us call $t_{in}$ and $t_{out}$ the times at which a particle respectively enters and exits the FoV. Then, let us call $\tau _{start}$ and $\tau _{end}$ the start and end times of the camera exposure. Thus, the conditions for capturing the signal of a particle in the FoV are $t_{in} < \tau _{end}$ and $t_{out} > \tau _{start}$. We can substitute $t_{out} = t_{in} + \text {FoV}/v$, being $\text {FoV}/v$ the time that a particle takes to travel through the FoV, obtaining $\tau _{start} - \text {FoV}/v< t_{in} < \tau _{end}$. Since the density of particles in the mixture is quite low, we can consider the passage of particles as independent events. Therefore, the process of imaging the pattern from *k* particles in the FoV can be considered as the Poisson process describing the occurrence of *k* events $t_{in}$, with a time rate $R_f$, in a time interval $\tau +\text {FoV}/v$, with probability:

5 $$\begin{aligned} Pr(k,\tau +\text {FoV}/v,R_f) = \frac{\left[ R_f(\tau +\text {FoV}/v) \right] ^k}{k!}e^{-R_f(\tau +\text {FoV}/v)} \end{aligned}$$

In our case $\tau ={29}\,\upmu \text {s}$ and we can calculate $R_f$ by multiplying the flux rate (0.2 ml/min) by the estimated particle concentration, which depends on the particle class ($1.6\times 10^4$ and $0.91\times 10^4\frac{\rm {particles}}{\rm {ml}}$ respectively for class A and B) since the mixtures have a common solid content volume. Note that we are assuming that the number of particles that remain stuck somewhere before reaching the illumination area is negligible w.r.t. the total number of passing particles. Therefore, even if we deem this assumption sufficiently true in our case, we should keep in mind that the estimated $R_f$ is more an upper limit for the true particle flow rate. From the next calculation steps it will be evident that this implies that we will obtain a lower limit estimate of the true FoV. To provide an example calculation, assuming a reasonable $\hbox {FoV}={100}\,\upmu \text {m}$, respectively for classes A and B we obtain (keeping 2 significant digits): $Pr_A(k=0) = 0.98$, $Pr_B(k=0)=0.99$, $Pr_A(k=1) = 0.017$, $Pr_B(k=1)=0.0098$, $Pr_A(k=2) = 0.00016$, $Pr_B(k=2)=0.000048$. These results are qualitatively consistent with both our visual checks and our assumption that the particles do not significantly often interact during their passage through the microfluidic channel (statistical independence). The particle ratio *R* can be estimated by $R=1-Pr(0,\tau +\text {FoV}/v,R_f)$, with reference to equation (5). Thus, by inverting it, we can finally estimate the FoV corresponding to a chosen value of *R*:

6 $$\begin{aligned} \text {FoV}=-\frac{\text {ln}(1-R)v}{R_f}-\tau v \end{aligned}$$

For each chosen value of *R* and for each particle class, we report in Table 3 the number of classification samples (accepted images) and the FoV estimates. The corresponding estimated FoV is quite large: $\sim {0.3}\,\text {mm}$. It should also be stressed that, as a consequence of our choice of having a single threshold $\theta _P$ for both classes and for training and testing, the FoV was class-dependent.

**Table 3:** Correspondence between chosen particle ratio *R* values (same for both particle classes), the number of images accepted as samples for classification (with strong enough particle signal) and estimated FoV of the classification process. *Left* and *right* tables regard respectively the configurations with and without a diffraction grating interposed between the microfluidic channel and the camera (*NDG* and *DG* configurations).

Particle rate	# accepted images		Field of view (mm)		Particle rate	# accepted images		Field of view (mm)	
	class A	class B	class A	class B		class A	class B	class A	class B
No diffractive layer					Diffraction grating				
0.02	1427	2108	0.09	0.25	0.02	1416	2288	0.08	0.27
0.04	4008	3067	0.27	0.37	0.04	4173	3213	0.27	0.38
0.06	6452	4120	0.45	0.51	0.06	6826	4207	0.45	0.51
0.08	7954	6051	0.56	0.76	0.08	8354	6199	0.57	0.76

## Data Availability

The datasets generated during the current study are available from the corresponding author on reasonable request.
